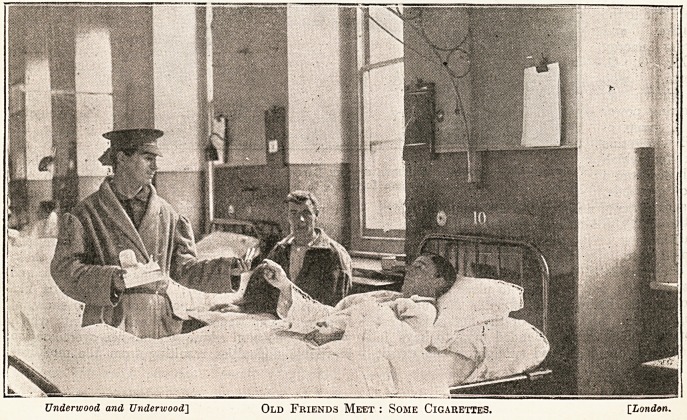# The Hospital Life of the Wounded: Comradeship, Cheerfulness, and Cigarette-Smoking

**Published:** 1915-06-12

**Authors:** 


### THE HOSPITAL LIFE OF THE WOUNDED.

### Comradeship, Cheerfulness, and Cigarette-Smoking.

We have great pleasure in giving this week three illustrations which, in addition to two appearing in the Hospital Sunday section (pp. 233-238), bring forcibly to mind the actual life lived by the wounded in the voluntary and other hospitals throughout the country. The comradeship which prevails between the wounded of all sections of the Navy and Army and the convalescent and unwounded members of the Services outside is well illustrated by these photographs. The scenes depicted in the illustrations to this article occurred, or rather occur daily, at St. Thomas's Hospital, where the first of the new huts for wounded men was opened on Wednesday. These huts, of which a detailed description, with plans, is given elsewhere in this Number are there described as "amongst the best buildings of the kind to be met with anywhere." Their origin and success are very largely due to the initiative and untiring energy of Mr. G. Q. Roberts, who has shown an organising power and a grasp of detail in this work for which we all owe him a debt of gratitude. The suggestion of utilising the site came from him, and the ability to tackle successfully the difficulties of making use of it efficiently is mainly his also. Hospital men have taken a magnificent part in that organisation of the nation which the war has thrust upon us, and it is well to place on record a few out of the many typical services they are rendering at this time. One of the first and most important things for a wounded man to have in abundance is fresh air and sunlight. The extent to which this is rendered possible by a great hospital of modern construction is well illustrated by the photograph recording a scene on the balcony. This shows the absolute satisfaction and happiness which dominates the mind of our soldiers and sailors when they find themselves at home in the hospital. All these pictures are well worthy of close study, and none probably deserves it more than that entitled "A Scene on the Balcony."

*Underwood and Underwood*] A Scene on the Balcony. [*London.*
"We are fortunate to be here."

Comradeship is further illustrated in the very interesting photograph of a ward in which two wounded soldiers are spending a quiet hour together whilst they make steady progress on the high-road to recovery. We have heard much of the incessant demand for cigarettes, which has been one of the features of the many efforts made to alleviate and help the life of the sailor and the soldier when engaged on service. Here, then, is a practical record, very suggestive to those who have the art of reading illustrations as carefully as the surrounding text, of the wounded enjoying the gifts which the public has been delighted to give them in token of its regard for their services. The alleviations of convalescence, and the spirit of cheerfulness and friendship which animates the soldier, are here seen and recorded, and form an encouragement to those who have done their best inside our hospitals and out to contribute to them. These photographs, like those which appear on another page, also show, at least to the experienced eye, the success with which voluntary hospital administration has overcome the inevitable difficulties resulting from the modification of their ordinary *régime* through the reception of a very large number of men not only fresh from the battlefield, but accustomed to military life and discipline. In our illustration will be found a record of a visit paid by a comrade who is hale and well to his wounded pal in the hospital. Here cigarettes constitute a feature of the illustration, and seem to indicate the liberality of the giver, who is at the same time reasonably mindful of the many wounded soldiers in his neighbourhood who may each like to have a share. Indeed, one of the curious minor facts of the war which future hospital historians will note is the enormous amount of cigarette-smoking which is indulged in by the wounded, and which public sentiment has fostered to an almost unlimited degree. The prevalent note of these pictures is cheerfulness and comfort. The feeling we have found to prevail amongst the soldier patients in our great hospitals everywhere has been one of recognition and gratitude for the good fortune which places them when wounded in the wards of a great hospital. It is well to remind the public, at all events, that this spirit of gratitude is the voluntary hospitals' reward for the tradition of consideration for the patients that they have built up through long years of peace. For this gratitude means that the spirit of the voluntary hospitals has so permeated their work that every class of patient regards it as good fortune to be in a hospital. We ask no better tribute.

*Underwood and Underwood*] [*London.*

THE HIGH ROAD TO RECOVERY. A QUIET HOUR.

*Underwood and Underwood*] OLD FRIENDS MEET : SOME CIGARETTES. [*London.*

## Figures and Tables

**Figure f1:**
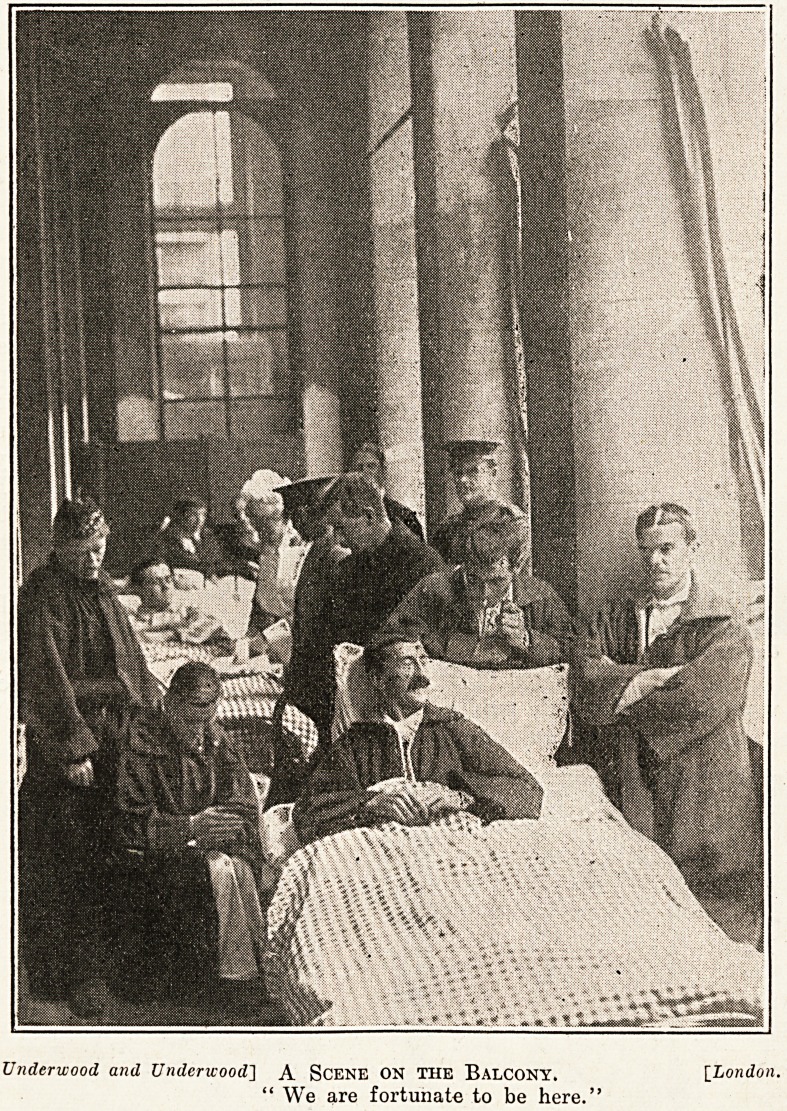


**Figure f2:**
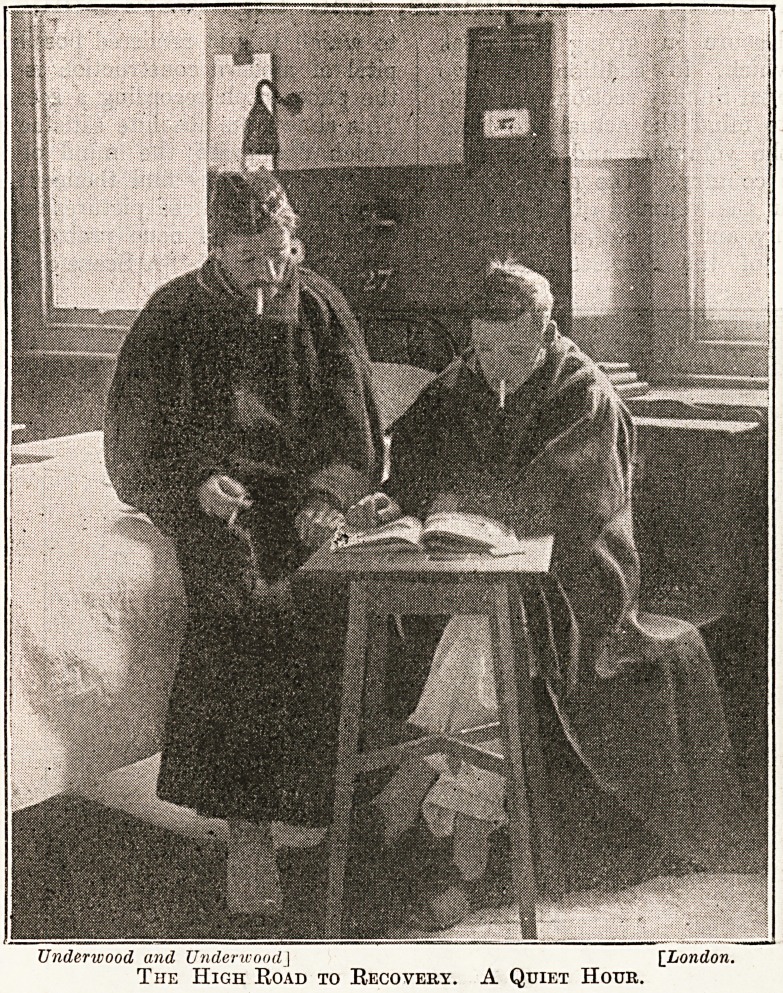


**Figure f3:**